# A Rare Case of Localised Isolated Penile Fournier's Gangrene and a Short Review of the Literature

**DOI:** 10.1155/2018/5135616

**Published:** 2018-05-09

**Authors:** Antonios Katsimantas, Nikolaos Ferakis, Panagiotis Skandalakis, Dimitrios Filippou

**Affiliations:** ^1^Department of Urology, Korgialenio-Benakio Hellenic Red Cross Hospital, Athanasaki 1, 11526 Athens, Greece; ^2^Department of Anatomy and Surgical Anatomy, Medical School, National and Kapodistrian University of Athens, Athens, Greece

## Abstract

Penile Fournier's gangrene (FG) is very rare clinical entity, which is also known as penile necrotizing fasciitis or wet gangrene of the penis. It is associated with increased morbidity and mortality and in the majority of the described cases it affects not only penis but also the adjacent organs and tissues (e.g., bladder, muscles, rectum, testis, and scrotum). We report a rare case of a previously healthy 68-year-old male, who presented with acute isolated penile Fournier's gangrene. Pus culture was identified with pathogens* Enterococcus faecalis*,* Streptococcus gordonii*, and* Prevotella melaninogenica*. Prompt surgical exploration, fluid resuscitation, antibiotic treatment, and diligent postoperative care are the cornerstone in the successful treatment of this emergency with high mortality.

## 1. Introduction

In 1863 JA Fournier, a French venereologist, described five cases of penis and scrotum gangrene. Since then more than 500 cases have been reported in the literature [1]. Penile Fournier's gangrene (FG) is very rare clinical entity, which is also known as penile necrotizing fasciitis or wet gangrene of penis [2, 3]. Fournier's gangrene is a rare rapidly progressing and potentially fatal soft tissue synergistic infection of the genitalia, perineum, perianal region, and abdominal wall [1]. Several comorbidity factors have been associated, as previous operation in the area or injury, diabetes mellitus, lupus erythematosus, and immunosuppression [1].

We present an uncommon case of spontaneous, isolated, penile necrotizing fasciitis in a previously healthy patient. Our aim is to underline the significance of early intervention in order to avoid extensive tissue destruction, sepsis, and death.

## 2. Case Report

A 68-year-old male patient presented with a two-day history of a swollen, painless penis, fever (up to 38.7°C), and malodorous discharge from his preputial ring. His medical history included inguinal hernia repair five years ago. The patient denied any recent history of trauma, voiding symptoms, alcohol abuse, and diabetes mellitus or other systemic disease. He had no sexual intercourse during the last nine years, since his wife's death. Patient's children confirmed his history's information.

**Figure 1:**
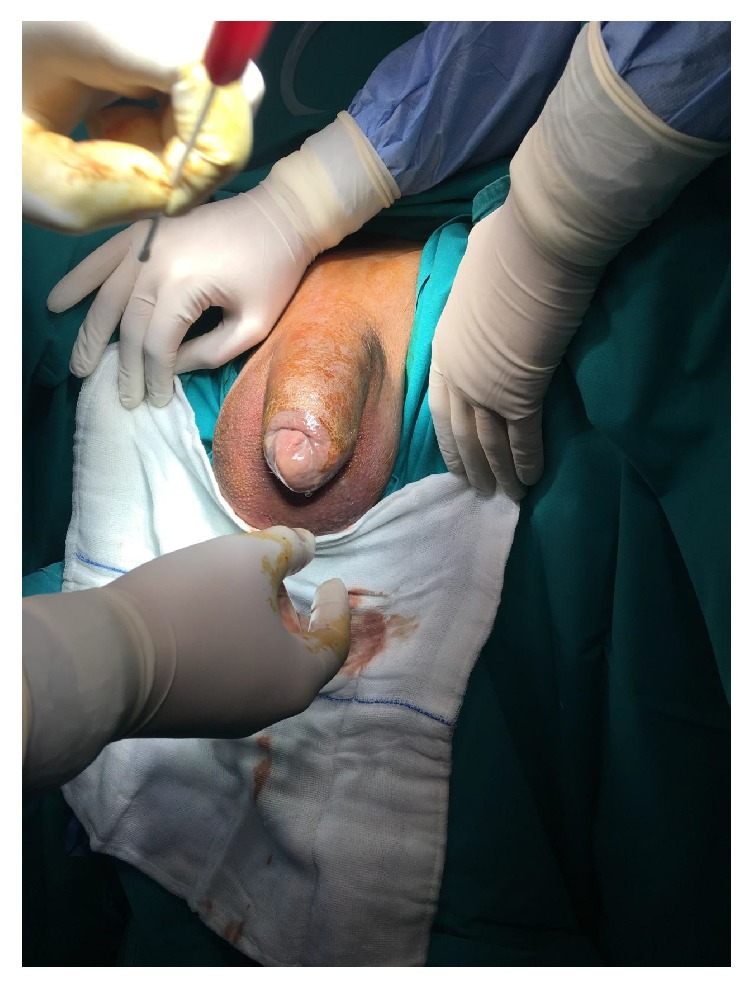
Swollen and dark-coloured penile shaft and purulent discharge from preputial ring.

On admission, his temperature was 38.4°C and the vital signs were stable. Clinical examination revealed edema, tenderness, and diffuse crepitus along the penile shaft. The penile skin was dark-coloured, but there are no other abnormal findings. There was malodorous, thick, purulent discharge from his preputial ring and we could not retract the foreskin (Figure 1). The scrotum, digital rectal, and inguinal lymph node examinations were normal.

**Figure 2:**
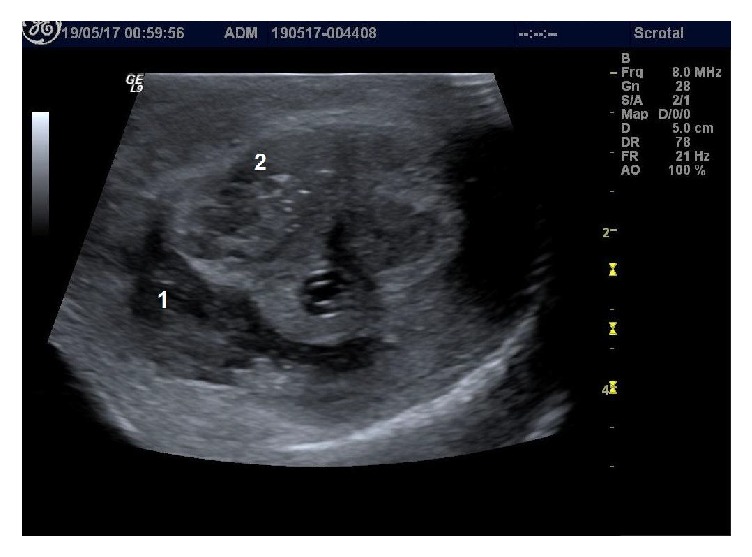
Preoperative ultrasonography of the penile shaft demonstrating hyperechogenic fluid collection with gas at the middle of the penile shaft, in contact with the right corpus cavernosum and corpus spongiosum (1) and gas in the right corpus cavernosum (2).

White blood cell count was elevated (11.9 × 10^3^/*μ*L), hemoglobin value was within normal levels (11.2 g/dL), and C-reactive protein value was found to be highly elevated (182 mg/L with normal values < 3 mg/L). The other blood chemistry levels and urinalysis were in the normal range. HIV test and RPR test for syphilis were negative. Ultrasonography demonstrated a 3 cm hyperechogenic fluid collection with gas at the middle of the penile shaft, in contact with the right corpus cavernosum and corpus spongiosum. Moreover, there was gas in the right corpus cavernosum and hyperemia of the surrounding tissue (Figure 2). The above-mentioned findings suggested the possibility of polymicrobial necrotizing infection.

Blood, urine, and pus cultures were obtained. Fluid resuscitation and antibiotic treatment with IV clindamycin, piperacillin-tazobactam, and vancomycin were administered, according to the Internal Medicine consultation.

**Figure 3:**
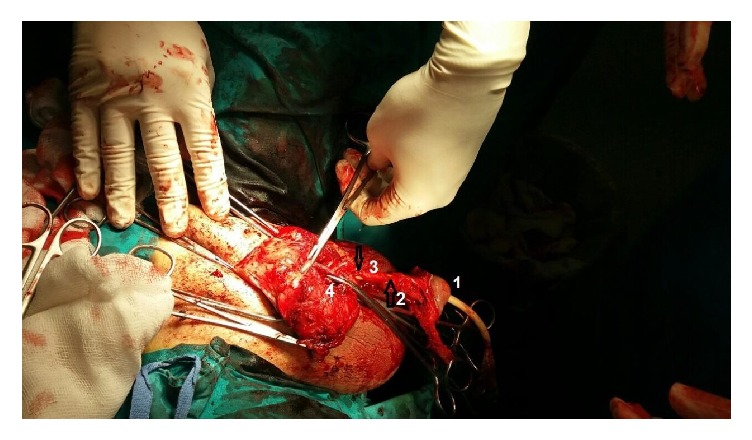
Intraoperative photo demonstrating normal, well-vascularised glans (1), corpus spongiosum (2), penile urethra (3), and clamped right cavernosal artery (4). There are two points on penile urethra which were debrided due to infection (arrows).

The patient submitted to urgent surgical intervention under general anesthesia. Before the operation, a suprapubic tube and a 20-Fr Foley catheter were inserted. After circumcision and degloving of the penis, it was noticed that although glans appeared normal both cavernosal bodies were replaced by necrotic tissue and pus up to their middle. No blood clots were found but only partial expansion of the inflammation to corpus spongiosum and urethra. Both testicles were normal. Necrotic tissue was debrided to bleeding edges. Glans was still well-vascularised, despite ligation of dorsal arteries of the penis and of cavernosal arteries. However, partial penectomy was performed mainly due to the partial excision of corpus spongiosum and urethra (Figure 3). Samples were collected for culture and histopathological examination.

**Figure 4:**
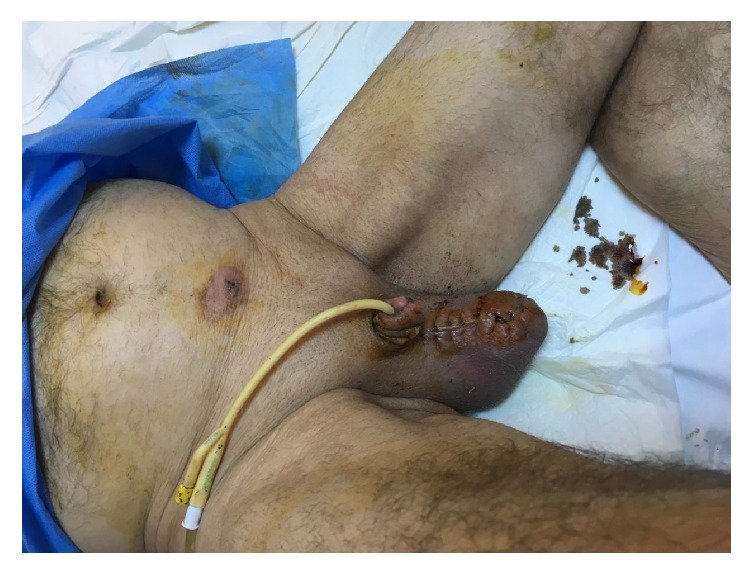
Penis after the closure with skin flaps.

Postoperatively, the patient remained afebrile. Pus culture suggested* Enterococcus faecalis*,* Streptococcus gordonii*, and* Prevotella melaninogenica*. Clindamycin was replaced by metronidazole according to the antibiotics' sensitivity diagram. Histology revealed acute and chronic inflammation and necrotic tissue. After repeated debridement and dressings twice a day, the penile stump improved at the 11th postoperative day. Penile skin flap was reapproximated over the surgical trauma's surface and the urethra was spatulated and approximated to the penile skin with sutures (Figure 4). The patient was discharged on the 17th postoperative day. The Foley catheter was removed on the 20th postoperative day and the patient is able to void in standing position.

## 3. Discussion

Fournier's gangrene is a rapid and fulminant polymicrobial infection of the fascia, with secondary necrosis of the subcutaneous tissues [1, 4, 5]. Its mortality rate is high [3, 5]. FG affects the genital region, may affect perineal and perianal regions, and is rarely limited to the penis [1–5]. It appears more frequently among males and elderly [1, 2, 5].

Both aerobic and anaerobic microorganisms may be implicated in the infection [1, 2, 4, 5]. Cultures usually reveal* Escherichia coli*,* Streptococcus*,* Staphylococcus*,* Enterococcus*, and* Bacteroides* [2, 4]. Their origin is usually diseases, trauma, or iatrogenic injury of urogenital and colorectal regions, but many cases are idiopathic [1–5]. In our case,* Enterococcus faecalis*,* Streptococcus gordonii*, and* Prevotella melaninogenica* were isolated in pus culture. However, we did not recognize the source of infection by patient's history, clinical evaluation, radiologic examinations, and intraoperative findings.

Predisposing factors for FG are diabetes mellitus (most common), obesity, cancer, alcohol abuse, advanced age, poor hygiene, malnutrition, heart and peripheral arteries diseases, liver disease, renal failure, HIV infection, and immunodeficiency [1–5]. Penile FG may originate from superinfection of dry gangrene of the penis [3]. Penile dry gangrene is the result of ischemia and is most commonly associated with end-stage renal disease and long-standing diabetes mellitus [1, 2]. It can be managed conservatively or surgically [1, 2]. No predisposing factors have been identified in the present case.

As it was already mentioned previously, glans was well-vascularised intraoperatively despite ligation of dorsal arteries of the penis. The dorsal artery provides most of the blood supply to the glans, which is also supplied by the bulbourethral artery [6]. Keeping urethra and glans may help to improve patient's quality of life. However, under particular circumstances as was in the present case, being more aggressive may be useful because there may be parts of corpus spongiosum and urethra affected by the infection.

Diagnosis is mainly based on history and clinical examination, but the physician should be experienced to suspect this rare pathology [5]. The most common clinical signs and symptoms are pain, edema, necrosis, foul odor, and crepitation on palpation, accompanied by fever [3–5]. X-rays, ultrasonography, computed tomography, and magnetic resonance imaging may contribute to the immediate and accurate diagnosis [5].

Treatment consists of rapid and aggressive surgical debridement of the necrotic tissue to bleeding edges (partial or total penectomy) under general or spinal anesthesia, suprapubic catheter insertion, removal of foreign bodies, and fluid resuscitation [1–5]. Broad-spectrum antibiotics are given empirically and according to the result of the cultures [2, 4, 5]. Serial necrotic tissue debridement may be needed [4, 5].

In our opinion, wound care and debridement of necrotic tissues using surgical spoon, under opioid administration, at least twice a day are of paramount importance, prevent progression and need for further debridement under anesthesia, and improve prognosis. Adjuvant hyperbaric oxygen therapy can help in FG's treatment postoperatively [2].

Isolated penile FG is a rare disease which can affect men who are healthy and without predisposing factors. Early and aggressive intervention prevents progression of the disease, can be lifesaving, and can improve the quality of life [2, 3, 5].
